# Parental mental illness and the risk of offspring cancer in childhood: a pooled meta-analysis of English and Swedish national cohorts

**DOI:** 10.1186/s12888-025-07520-w

**Published:** 2025-10-27

**Authors:** Alicia Nevriana, Cemre Su Osam, Kyriaki Kosidou, Holly Hope, Darren M. Ashcroft, Susanne Wicks, Christina Dalman, Kathryn M. Abel, Matthias Pierce

**Affiliations:** 1 Department of Global Public Health, Karolinska Institutet, Stockholm, 171 77 Sweden; 2 Institute of Environmental Medicine, Karolinska Institutet, Stockholm, 171 77 Sweden; 3 Centre for Women’s Mental Health, School of Health Sciences, Faculty of Biology, Medicine and Health, University of Manchester, Jean McFarlane Building, Oxford Road, Manchester, M13 9PL UK; 4 Center for Epidemiology and Community Medicine, Region Stockholm, Sweden; 5 Centre for Pharmacoepidemiology and Drug Safety, School of Health Sciences, Faculty of Biology, Medicine and Health, University of Manchester, Jean McFarlane Building, Oxford Road, Manchester, M13 9PT UK; 6 NIHR Greater Manchester Patient Safety Research Collaboration (PSRC), University of Manchester, Manchester, M13 9PL UK; 7 Greater Manchester Mental Health NHS Foundation Trust, Bury New Road, Prestwich, Manchester, Greater Manchester, M25 3BL UK

**Keywords:** Mental disorders, Neoplasms, Parents, Child, Cohort

## Abstract

**Background:**

Parental mental illness’ effects on risk of childhood cancers is largely unknown. This study determined the association between maternal or paternal mental illness and risk of childhood cancers.

**Methods:**

Retrospective cohort studies and meta-analysis using population-based registers from England and Sweden. 591,092 children born 1996–2017 (England) and 2,192,476 children born 1991–2011 (Sweden) were linked to their mothers (both countries) and fathers (Sweden), followed until latest December 2016 (Sweden) or July 2017 (England). Parental mental illness (depression/anxiety, psychosis, alcohol/substance use disorders, eating/personality disorders) were identified through primary (England) or secondary care (Sweden) databases as time-varying exposure, measured from one year before birth until the end of follow-up. Childhood cancer were identified from secondary care data. Hazard ratios (HRs) were estimated using Cox models separately in both countries, adjusted for potential confounders. Random-effects meta-analyses were used to estimate the association for maternal exposure.

**Results:**

All estimates were characterised by uncertainty, with 95% confidence intervals including scenarios where there was no association. The point estimate represented an increased risk of childhood cancer associated with maternal alcohol or substance use disorders; however, the 95% confidence interval narrowly crosses the null, therefore, this cannot be inferred with certainty (pooled HR 1.30, 95% CI 0.97–1.75). Conversely, point estimates suggest a decreased risk of childhood cancer associated with maternal psychosis (HR 0.76, 95% CI 0.53–1.09) and paternal depression/anxiety (HR 0.85, 95% CI 0.71–1.02); however, these confidence intervals also cross one, so a null effect—or even a small increased risk—cannot be fully ruled out.

**Conclusions:**

There was some tentative evidence of an association between parental mental illness and childhood cancer; however, the uncertainty in the estimates precludes strong conclusions. Further data may clarify these associations.

**Supplementary Information:**

The online version contains supplementary material available at 10.1186/s12888-025-07520-w.

## Background

Each year, globally, around 400,000 children and adolescents are diagnosed with cancer [1] and 130,000 die [2], with the incidence continuing to rise [1, 3]. Compared to cancer in adults, childhood cancer usually progress more rapidly [4] and survivors might experience adverse health outcomes that last well into adulthood [5]. Nevertheless, little is known about causes of childhood cancer [6].

An estimated 10–25% of children live with a parent experiencing mental illness [7, 8] and they are more likely to have poor physical health, including atopic illnesses [9], injuries [10], and missing vital vaccinations in children [11]. However, less is known about the possible effects of parental mental illness on relatively rare childhood diseases like cancers [9].

A clue to this association might be seen in studies of mental illness and subsequent cancer risk in individuals and first-degree relatives. A meta-analysis reported that people with schizophrenia have a reduced risk of developing cancers, although the risk varied by sex and cancer types [12]. However, another meta-analysis among women [13] and a later cohort study [14] found an increased risk of cancer for people with schizophrenia. Increased risks for cancer have also been reported among individuals with substance use disorders [14–16]. However, for depressive disorders, some studies found increased risks overall [14, 17], while others found only increased risk for specific cancer types [18]. On the other hand, for anxiety/stress-related disorders, a study found an increased risk [14], while two studies found no association [18, 19]. For eating disorders, one study found an increased risk [14], while another study found a slightly decreased risk of cancer among women but not men [20]. Only one study assessed the risk for individuals with personality disorders and this study found an increased risk [14]. Familial aggregation studies were currently limited to people with schizophrenia, and these studies showed reduced risks of cancers among their first-degree relatives [21, 22]. A range of hypotheses has been proposed for these varying associations, including the presence of confounders, such as smoking or diet [12, 13, 15–17], the use of medications that affect hormonal axes, such as antipsychotics [12, 13], or shared genetics [17].

Whether these results extend to children of parents with mental illness is unclear. One Danish study found similar rates of cancer among adult offspring of parents with schizophrenia, bipolar disorders, or depression compared to offspring without parental mental illness [23]. Some studies showed increased risk for childhood cancer associated with maternal alcohol or substance use [24–26] but studies including paternal effects were scarce [24]. It has been suggested that shared genetic factors [23] or epigenetic mechanism resulting from parental substance use disorders might have a causal role in childhood cancer [24–26]. Another potential mechanism might be through increased risks from health behaviours that contribute to cancer development (e.g., smoking), which are more common among offspring with parental mental disorders [27].

Previous studies might have been underpowered to detect possible associations between parental mental illness and childhood cancer, because of the rarity of both exposures and outcomes. We address this problem by conducting parallel analyses of large population-based cohorts in England and Sweden and pooling results using meta-analyses. This provided us with sufficient numbers of cases to examine the effects of maternal vs paternal mental illness, overall and by illness subtype. We thus aim to quantify, in the most detailed study to date, the association between different types of maternal and paternal mental illness and the risk of childhood cancers.

## Methods

### Study design and settings

Two retrospective cohort studies were conducted using registers from England and Sweden from overlapping periods. Data for the English cohort were extracted from the Clinical Research Practice Datalink (CPRD-GOLD), comprised of anonymised primary care health records from approximately 10% of the UK population. In the UK, 98% of the population is registered with a general practice [28] and most mental illness is managed within primary care [29]. The CPRD contains data on patient’s clinical consultations, prescriptions and external healthcare referrals. Study participants were identified from the CPRDs mother-baby link [11], which is an algorithm developed within CPRD to identify mother-baby pairs [30]. Information on fathers could not be identified from the CPRD. To ascertain hospital inpatient records, children in this cohort were linked to Hospital Episodes Statistics (HES) dataset using a unique NHS identifier. Linkage to the HES dataset is only available on practices that consented to linkage (75% of English practices).

Children in the Swedish cohort were identified from the Total Population Register, which also contains demographic information. Children were linked using a unique personal identification number (assigned to all Swedish residents) to parents using the Multi-generation Register; parental mental illness exposure using the National Patient Register (NPR), which contains information on inpatient and specialised outpatient care; cancer outcomes using the Swedish Cancer Register; individual and family-level socioeconomic information using the Longitudinal integrated database for health insurance and labour market studies (LISA); and perinatal characteristics using the Medical Birth Register.

### Study population

**Fig. 1:**
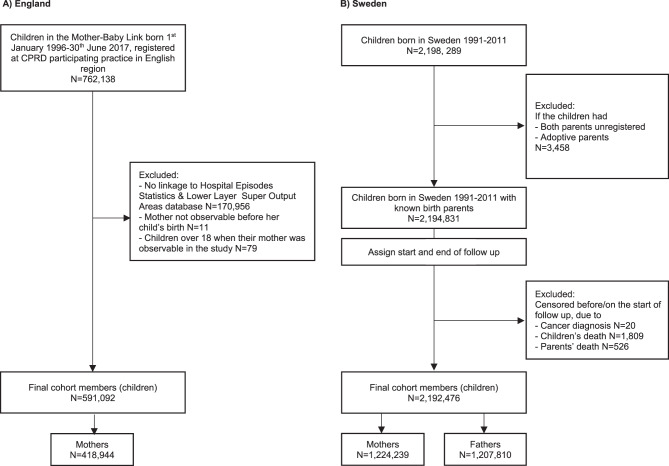
Cohort selection **A**) England, **B**) Sweden

For the English cohort, eligible children were selected from all those born on 1^st^ January 1996–30^th^ June 2017 and linked to HES. We excluded those whose mother was registered at a general practice for less than six months before the child’s birth (*n* = 171,046; Fig. 1A). This was because data were not captured before registration and 6 months was considered sufficient follow-up to capture exposure status. Children’s follow-up started at the latest date of: their birth date; they were registered at the clinical practice; when the practice began submitting data to the CPRD; or when HES linkage began (1^st^ April 1997). Children’s follow-up ended at the earliest date of: transfer out of clinical practice; 18^th^ birthday; death; their mother’s death; end of data collection by the practice; or the study end (31^st^ July 2017). In total, 591,092 children and 418,944 mothers were identified for analysis with a mean follow-up time of 11.2 years.

For the Swedish cohort, eligible children were those born in Sweden in 1991–2011. We did not include children who were born outside Sweden since we were less likely to have information on parental health and socioeconomic information prior to childbirth within the registers among these children. Children were excluded if both parents were unregistered or adoptive (*N* = 3,458; Fig. 1B). Children’s follow-up started from their birthdate until the earliest date of: emigration or death of either parent or children; their 18^th^ birthday; or end of data collection (31^st^ December 2016). The Swedish cohort consisted of 2,192,476 children linked to 1,244,239 mothers and 1,207,810 fathers, with a mean follow up time of 13.2 years.

### Maternal and paternal mental illness

Maternal and paternal mental illness was a time-varying exposure where the children in both cohorts were considered unexposed until the first record (date) of mental illness, where they would be deemed exposed until the end of follow-up. We included records of common mental illness (depression or anxiety), psychotic disorders (non-affective or affective psychosis), alcohol/substance use disorders, and other mental illness (eating disorders, personality disorders) as defined below.

In the English cohort, maternal mental illness was identified using data recorded in primary care. Diagnoses are not necessarily repeatedly recorded in a patient’s primary care healthcare record; furthermore, for anxiety and depression, healthcare professionals are increasingly recording symptoms (e.g., ‘low mood’) [31, 32] and prescribing rather than recording a diagnosis. Therefore, to capture discrete episodes of mental illness we used an algorithm that triangulated four pieces of information: mental illness diagnosis, referral to psychiatric care services, psychotropic medication, or symptoms. A diagnosis or referral were sufficient to identify an episode of mental illness. For psychotropic medications or symptoms, there needed to be a related historical diagnosis, or a psychotropic medication and a related symptom within three months. For example, to be identified as having depression, a patient with the symptom ‘low mood’ needed to have either a historical depression diagnosis or a prescription for an antidepressant within three months of the recorded symptom. Two psychiatrists (KMA and ES) reviewed the code lists and allocated psychotherapeutics and symptoms to mental illness diagnoses [7]. Evidence from a UK study that combined a national survey with primary care data demonstrated that people ascertained to have a mental illness from their primary care record, using a similar mental illness algorithm to the authors’, also scored low enough on a validated mental health measure to indicate caseness for mental illness, indicating the validity of identifying “true” cases of mental illness from the primary care record [33]. Data on paternal mental illness was not available for this cohort.

In the Swedish cohort, first recorded diagnosis of maternal and paternal mental illness was identified from the NPR using ICD codes (Table S1).

### Childhood cancer

In the English cohort, childhood cancer diagnosis was captured from the child’s inpatient admission records using ICD-10 codes (C00–C97; Table S2). In the Swedish cohort, childhood cancer was obtained from the Swedish Cancer Register using ICD-9 codes (140–208).

### Covariates

We extracted information on factors that are related to mental disorders among parents [34–36] and childhood cancer [37–39]. In both cohorts, we extracted information on child’s sex, birth year, parental age at birth, and maternal smoking. In the English cohort, we additionally extracted information on maternal antibiotic use during pregnancy, child’s ethnicity, and area-level measure of deprivation based on the 2010 quintile of the Index of Multiple Deprivation of the residential address. In the Swedish cohort, we additionally extracted information on parental country of birth (Sweden/other countries), parental history of cancer, parental education, and household disposable income. Parental education was defined as the highest educational attainment of the mothers or fathers at childbirth, categorised into compulsory (≤9 years), secondary (10–12 years), and university education (≥13 years). Household disposable income was defined as the yearly sum of income and public benefits earned by all family members after taxes, categorised into quintiles for each calendar year.

### Statistical analysis

Crude and adjusted hazard ratios (HR and aHR) for the association between maternal or paternal mental illness and time to incident childhood cancer were estimated using the Cox proportional hazard model. For the English cohort analysis, the adjusted model included: maternal age at birth, maternal smoking, maternal antibiotic use during pregnancy, maternal comorbid mental disorders, deprivation, region, child’s sex and ethnicity. For the Swedish analysis, the adjusted model included: child’s sex and birth year, parental country of birth and age at childbirth, parental education, household disposable income, parental history of cancer, and parental comorbid mental illness. Maternal smoking was not included in the adjustment for the Swedish cohort since the data was only available for children born 1990–2010 (*N* = 2,018,555). In both cohorts, clustering by maternal or paternal sibships was accounted for using the Hubber/White sandwich estimate of the standard error [40]. In the English cohort, we excluded missing observations (43 children). In Swedish cohort, missing observations were included as a separate category.

Pooled estimates (aHRs) were calculated using random-effects meta-analysis. Heterogeneity was evaluated using I^2^ and τ^2^ statistics [41]. Hazard Ratios in both cohorts were estimated using Stata 16 and meta-analysis was carried out in R (Version 4.2.3) using ‘meta’ [42] and ‘metafor’ [43] package.

## Results

### Cohort characteristics

Across both cohorts, 2,783,568 children were followed up for 34,993,431 person-years. Mean maternal age was 30 years (standard deviation (SD): 5.8 for UK, 5.2 for Sweden) and 49% of children were female (Table 1).

**Table 1:** England (*N* = 591,092) and Sweden (*N* = 2,192,476) cohort demographics and socioeconomic characteristics by parental mental illness exposure

Variables	England		Sweden	
	Children exposed to any maternal mental illness	Children unexposed to any maternal mental illness	Children exposed to any maternal or paternal mental illness	Children unexposed to any maternal or paternal mental illness
	*N* = 205,804	*N* = 385,288	*N* = 421,887	*N* = 1,770,589
	n (%)	n (%)	n (%)	n (%)
**Follow up time (year)**				
Mean (SD)	11.9 (4.7)	10.7 (5.0)	13.7 (4.4)	13.0 (5.0)
**Child sex**				
Female	99,933 (48.6)	188,439 (48.9)	204,152 (48.4)	861,747 (48.7)
Male	105,871 (51.4)	196,849 (51.1)	217,735 (51.6)	908,842 (51.3)
**Birth year**				
1991–1995	-	-	100,835 (23.9)	477,537 (27.0)
1996–2001	56,885 (27.6)	86,855 (22.5)	120,359 (28.5)	426,936 (24.1)
2002–2007	69,812 (33.9)	118,700 (30.8)	125,974 (29.9)	490,012 (27.7)
2008–2013	63,648 (30.9)	137,839 (35.8)	74,719 (17.7)	376,104 (21.2)
2014–2017	15,459 (7.5)	41,894 (10.9)	-	-
**Child ethnicity**				
White	176,751 (85.9)	287,163 (74.5)	-	-
Other	15,336 (7.4)	63,201 (16.5)	-	-
Unknown/missing	13,717 (6.7)	34,924 (9.1)	-	-
**Parental country of birth**				
All parents born in Sweden	-	-	299,591 (71.0)	1,337,130 (75.5)
≥1 parent(s) born outside Sweden	-	-	122,295 (29.0)	433,410 (24.5)
Missing	-	-	1 (0.0)	49 (0.0)
**Maternal age at delivery**^**a**^				
< 20	10,636 (5.2)	13,791 (3.6)	12,138 (2.9)	17,578 (1.0)
20–29	94,213 (45.8)	148,481 (38.5)	206,582 (49.0)	779,027 (44.0)
30–39	92,633 (45.0)	204,938 (53.2)	187,197 (44.4)	910,371 (51.4)
> 40	8,322 (4.0)	18,078 (4.7)	15,921 (3.8)	63,082 (3.6)
**Paternal age at delivery**^**b**^				
< 20	-	-	3,646 (0.9)	4,289 (0.2)
20–29	-	-	141,965 (33.7)	496,736 (28.1)
30–39	-	-	212,595 (50.4)	1,012,224 (57.2)
> 40	-	-	59,932 (14.2)	232,546 (13.1)
**Parental education**				
Compulsory	-	-	46,533 (11.0)	88,860 (5.0)
Secondary	-	-	218,123 (51.7)	775,845 (43.8)
University	-	-	148,514 (35.2)	871,675 (49.2)
Missing	-	-	8,717 (2.1)	34,209 (1.9)
**Quintiles of deprivation**^**c**^				
Q1 (most deprived)	56,989 (27.7)	87,177 (22.6)	83,858 (19.9)	253,601 (14.3)
Q2	42,984 (20.9)	81,628 (21.2)	122,424 (29.0)	463,415 (26.2)
Q3	39,272 (19.1)	73,084 (19.0)	99,576 (23.6)	431,852 (24.4)
Q4	37,535 (18.2)	76,654 (19.9)	64,373 (15.3)	343,518 (19.4)
Q5 (least deprived)	29,024 (14.1)	66,745 (17.3)	47,142 (11.2)	261,235 (14.8)
Missing	-	-	4,514 (1.1)	16,968 (1.0)

^a^Including children with known mothers (Sweden)

^b^Including children with known fathers (Sweden)

^c^Based on household disposable income (Sweden) or UK Index of Multiple Deprivation of the general practitioner location (England)

In both cohorts, the most common maternal mental illness diagnosis was depression or anxiety (Table S5), although considerably more children in England had mothers diagnosed with depression or anxiety compared to Sweden (34.8% vs 12.6%). Contrastingly, more children in the Swedish cohort had mothers with psychotic disorders compared to England (1.6% vs 0.7%). In Sweden, 9% of children had fathers with any mental illness; the majority were diagnosed with depression or anxiety (7.3%); followed by alcohol/substance use disorders (2.8%).

The prevalence of maternal smoking in England was 23.6% for mothers without mental illness, however, this number was even higher for mothers with mental illness (46.7%; Table S3). In Sweden, the prevalence of maternal smoking was 10.0% and 19.8% for children with and without maternal or paternal mental illness, respectively.

### Association between maternal or paternal mental illness and childhood cancer

Overall, there were 6,195 (England: 951; Sweden: 5,251) incident childhood cancers over follow-up with a mean age of diagnosis of 6.4 years (SD: 5.3) in England and 6.9 years (SD: 5.4) in Sweden. The incidence rate of cancer among children exposed to any maternal mental illness was 16.4 per 100,000 person-years in England and 17.6 in Sweden (Table 2). The cancer rate among children exposed to any paternal mental illness in Sweden was 15.7 per 100,000 person-years. In both cohorts, the most common cancer diagnosis was lymphoid and haematopoietic cancer, followed by eye, brain, and central nervous system cancer (Table S3).

**Table 2:** Risk of cancer among children with and without maternal mental illness in England and Sweden

Type of mental illness	England				Sweden			
	N cancer cases	Incidence rate per 100,000 person-years (95% CI)	Crude HR (95% CI)	**Adjusted HR (95% CI)**^**a**^	N cancer cases	Incidence rate per 100,000 person-years (95% CI)	Crude HR (95% CI)	**Adjusted HR (95% CI)**^**b**^
**Any mental illness**								
Unexposed	637	15.2 (14.1–16.4)	1	1	4,926	18.2 (17.7–18.7)	1	1
Exposed	314	16.4 (14.7–18.3)	1.13 (0.99–1.30)	1.10 (0.95–1.27)	319	17.6 (15.8–19.6)	1.04 (0.93–1.17)	1.02 (0.90–1.15)
**Depression or anxiety**								
Unexposed	642	15.2 (14.1–16.5)	1	1	4,956	18.2 (17.7–18.7)	1	1
Exposed	309	16.4 (14.7–18.3)	1.13 (0.99–1.30)	1.10 (0.95–1.28)	289	17.5 (15.6–19.7)	1.04 (0.92–1.17)	1.02 (0.90–1.16)
**Psychotic disorders**								
Unexposed	-^c^	15.6 (14.7–16.7)	1	1	5,212	18.2 (17.7–18.7)	1	1
Exposed	-^c^	6.3 (1.6–25.3)	0.42 (0.10–1.69)	0.39 (0.10–1.55)	33	14.9 (10.6–20.9)	0.87 (0.62–1.23)	0.80 (0.56–1.17)
**Alcohol/substance use disorders**								
Unexposed	938	15.5 (14.6–16.5)	1	1	5,207	18.1 (17.7–18.6)	1	1
Exposed	13	22.6 (13.1–38.8)	1.52 (0.88–2.64)	1.58 (0.91–2.76)	38	22.8 (16.6–31.3)	1.34 (0.97–1.84)	1.21 (0.86–1.71)
**Other mental illness**								
Unexposed	943	15.6 (14.6–16.6)	1	1	5,197	18.1 (17.6–18.6)	1	1
Exposed	8	15.2 (7.6–30.4)	1.00 (0.50–2.00)	0.85 (0.41–1.76)	48	22.9 (17.3–30.4)	1.34 (1.01–1.79)	1.28 (0.95–1.73)

^a^Adjusted for child’s sex, child’s ethnicity, maternal age, maternal smoking during child’s life, antibiotic use during pregnancy and region

^b^Adjusted for child’s sex, birth year, parental age, parental country of birth, parental education, household disposable income in quintiles, parental history of cancer, maternal comorbid depression or anxiety, maternal comorbid psychotic disorders, maternal comorbid other mental illness (including alcohol/substance use disorders, eating disorders, personality disorders), paternal comorbid depression or anxiety, paternal comorbid psychotic disorders, paternal comorbid other mental illness (including alcohol/substance use disorders, eating disorders, personality disorders)

^c^Data was not presented due to the low number of observations (*n* < 5)

**Fig. 2:**
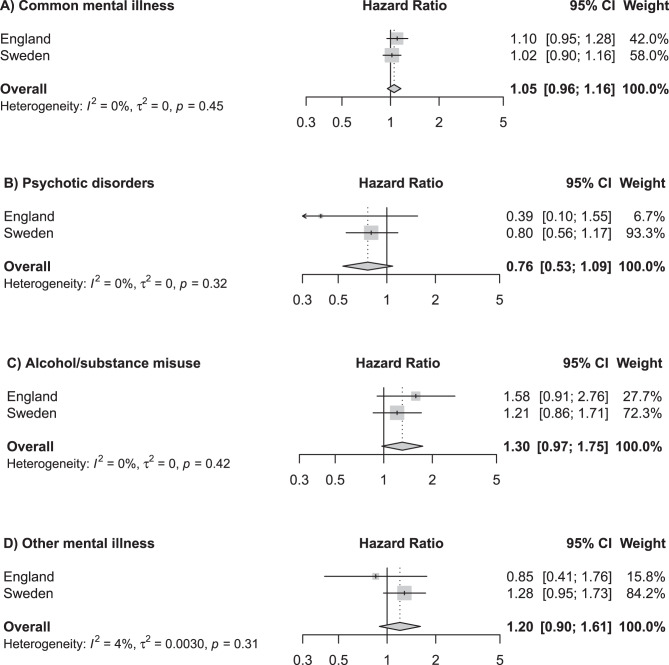
Results from pooled data analyses for the associations between maternal mental illness and childhood cancer

The estimate from the pooled analysis reported that maternal psychotic disorders was associated with a lower risk of childhood cancer, however, there was substantial uncertainty in this with the 95% confidence intervals including the scenarios where there was no association and where there was a small increase in risk (aHR 0.76, 95% CI 0.53–1.09, Fig. 2). On the other hand, maternal alcohol/substance use disorders was associated with an increased risk of cancer, although, again there was uncertainty in this (aHR 1.58, 95% CI 0.91–2.76). There was little evidence of an association between depression or anxiety and childhood cancers (Fig. 2).

In contrast with results for maternal exposure, exposure to paternal depression or anxiety from the Swedish cohort was associated with decreased risk of cancer, although there was uncertainty in this, with the 95% confidence interval marginally crossing 1 (aHR 0.85, 95% CI 0.71–1.02, Table 3). There was little evidence of a change in risk associated with paternal alcohol/substance use disorders.

**Table 3:** Risk of cancer among children with and without paternal mental illness in Sweden

Type of mental illness	N cancer cases	Incidence rate per 100,000 person-years (95% CI)	Crude HR (95% CI)	**Adjusted HR (95% CI)**^**a**^
**Any mental illness**				
Unexposed	4,998	18.3 (17.8–18.8)	1	1
Exposed	203	15.7 (13.7–18.1)	0.94 (0.82–1.09)	0.94 (0.81–1.09)
**Depression or anxiety**				
Unexposed	5,060	18.3 (17.8–18.8)	1	1
Exposed	141	14.1 (12.0–16.7)	0.85 (0.72–1.00)	0.85 (0.71–1.02)
**Psychotic disorders**				
Unexposed	5,170	18.1 (17.7–18.6)	1	1
Exposed	31	19.4 (13.6–27.5)	1.16 (0.81–1.65)	1.20 (0.82–1.75)
**Alcohol/substance use disorders**				
Unexposed	5,133	18.2 (17.7–18.7)	1	1
Exposed	68	17.3 (13.6–21.9)	1.04 (0.81–1.32)	1.00 (0.77–1.30)
**Other mental illness**				
Unexposed	5,185	18.2 (17.7–18.7)	1	1
Exposed	16	14.6 (8.9–23.8)	0.88 (0.54–1.44)	0.87 (0.53–1.44)

^a^Adjusted for child’s sex, birth year, parental age, parental country of birth, parental education, household disposable income in quintiles, parental history of cancer, maternal comorbid depression or anxiety, maternal comorbid psychotic disorders, maternal comorbid other mental illness (including alcohol/substance use disorders, eating disorders, personality disorders), paternal comorbid depression or anxiety, paternal comorbid psychotic disorders, paternal comorbid other mental illness (including alcohol/substance use disorders, eating disorders, personality disorders). Reference group is children without specific maternal or paternal mental illness diagnosis

## Discussion

This is the most comprehensive study to investigate the association of different types of maternal and paternal mental illnesses on the risk of children developing childhood cancer. Performing a meta-analysis in two national cohorts, we report that exposure to maternal psychotic disorders might be associated with a reduction in cancer risk in children by up to a quarter. There was borderline evidence of exposure to maternal alcohol/substance use disorders being associated with a slight increase in risk for childhood cancer. There was little evidence of an increased risk associated with other types of parental mental illness.

Previous studies have reported a reduced risk of cancer among first degree relatives of people specifically with schizophrenia, ranging from a 4–10% decrease in standardised incidence ratio for parents and 8–11% decrease in standardised incidence ratio for siblings [21, 22]. One possible explanation for this might be shared genetic effects. For example, the P53 gene (a tumour suppressor) has been reported to be associated with schizophrenia and reduced cancer risk [44–46]. This gene is suggested to increase cell death in vital areas of the central nervous system thought to have a role in the aetiology of schizophrenia [47]. The negative association that we observe here between maternal psychosis and subsequent childhood cancers is even more striking given the high rates of smoking among mothers with serious mental illness [48], and parental/maternal smoking is a known risk factor for childhood cancers [25]. Although we were able to control for reported smoking, this is relatively poorly measured and residual confounding is likely.

Nevertheless, in our study, we only observed reduced risk among children exposed to maternal, but not paternal psychotic disorders, although estimates were statistically non-significant. Whilst these discrepancies might be attributed to low power in the paternal analysis (only available in Sweden), this might also suggest that maternal effects involved in the control of fetal growth (including IGF-2 gene expression) also play a role in subsequent offspring cancer risk [49]. Indeed, approximately half of all childhood cancers are associated with birthweight [50] and maternal mental illness is well-established as a risk factor for poor fetal growth or small for gestational age [51]. Therefore, risks for certain childhood cancers may originate in utero e.g. following assisted reproduction [52] and in infants born large-for-gestation age [50]. On the other hand, this might also suggest that non-genetic mechanisms contribute to this association, for example through antipsychotic medication use, which may have anticancer properties [53].

A previous Danish registry study did not find an association between parental serious mental illness and offspring cancer risk (HR 1.00, 95% CI 0.96–1.04) [23]. However, there are some key differences between this and the current study. First, they combined maternal and paternal mental illness exposures. Second, they included depression as serious mental illness, whereas we grouped depression as common mental illness. Finally, they did not confine follow up to childhood but included incident cancer in adult offspring.

The suggested increased risk of cancer amongst children exposed to maternal alcohol or substance use disorders has been observed in other studies, although our findings were not statistically significant at the 5% level. One meta-analysis reported a 26% increased risk for neuroblastoma among children of mothers who reported alcohol use during pregnancy [25]. Another Canadian study reported a 4% increased risk of any cancer among children exposed to maternal illicit drug use before or during pregnancy [26]. However, another meta-analysis [24] found no evidence of a change in the risk of leukaemia (OR 0.98, 95% CI 0.84–1.14) among children exposed to maternal alcohol use during pregnancy. The increased risk of childhood cancer among children exposed to maternal alcohol/substance use disorders might be explained by teratogenic effects of alcohol, smoking and drugs [24, 25]. Exposure to alcohol and illicit drugs in utero might cause DNA damage [25, 54] and alter epigenetic programming [24, 25] which may play a role in the development of childhood cancers.

Despite these plausible explanations, it is important to acknowledge that discrepancies between our findings and those of previous studies may, in part, reflect limitations related to sample selection or publication bias. Additionally, given the rarity of both the exposure (parental mental illness) and the outcome (childhood cancer), our study remains underpowered to detect modest associations, as indicated by the wide 95% confidence intervals that almost uniformly include a null effect. These methodological constraints should be carefully considered when interpreting our results, particularly in relation to subgroup analyses.

One of the biggest strengths of this study is that we conducted a pooled analysis on a total of 2.7 million children, which allowed us to investigate a rare disorder. Also, by investigating two distinct populations with different data collection methods, the conclusions that could be drawn from any one population are strengthened and we might be able to generalise the results to a wider population. This collaborative study uses electronic health records from England and Sweden. Electronic health records significantly minimise the biases associated with prospective cohort studies which are usually in small sample sizes, and with selection biases resulting from loss to follow-up, as well as recall or self-reporting biases. Moreover, by using combinations of electronic health and administrative databases, we had access to a range of demographics, socioeconomic, and health variables that we could use when controlling for potential confounders.

However, there are some limitations. First, in both cohorts, some mental illnesses likely remained undetected because individuals did not seek help for illness. Second, in the UK, cancer diagnosis was retrieved from hospital inpatient data which was only available for England. This means that the cohort’s statistical power was reduced as there were no data from Scotland, Wales and Ireland. Moreover, even within England, we were unable to link to hospital data for 25% of primary care practices, which might result in selection bias. Additionally, because linkage in the UK data is based on childbirth health records, we were unable to link children with the fathers’ data. By contrast, in Sweden, we did not have access to primary care database, and so we might have missed some cases of parental mental illness treated exclusively within primary care. This might partially contribute to differences in the prevalence of maternal mental illness between England and Sweden, especially when it comes to more common and less severe problems such as depression or anxiety. However, this might also be viewed as strength, as we are able to triangulate findings across datasets with different population structures and different sources information. Nevertheless, although we have tried to control for all potential confounders, we still were not able to control for other potential confounders, including epi/genetic and environmental factors, such as exposure to pesticides, radiation and environmental pollution.

## Conclusion

Cancer affects hundreds of thousands of children globally and its incidence is rising, yet the aetiological mechanisms remain obscure. While our findings indicated no conclusive evidence on the effect of parental mental illness on childhood cancer, it should be noted that only a small proportion of the 95% confidence interval includes scenarios where there were null associations. More collaborative studies with even larger sample sizes might be needed to shed further light on the impact of parental mental illness on child’s health, including cancer risk.

## Electronic supplementary material

Below is the link to the electronic supplementary material.

Supplementary Material 1

## Data Availability

Read codes used are published on Clinicalcodes.org. Electronic health records are, by definition, considered ‘sensitive’ data in the UK by the Data Protection Act 2018, and cannot be shared via public deposition because of information governance restriction in place to protect patient confidentiality. Access to data are available only once approval has been obtained through the individual constituent entities controlling access to the data. The primary care data can be requested via application to the Clinical Practice Research Datalink (http://www.cprd.com/research); secondary care data can be requested via application to the Hospital Episode Statistics from the UK Health and Social Care Information Centre (https://digital.nhs.uk/data-and-information/data-tools-and-services/data-services/hospital-episode-statistics). The Swedish data cannot be shared publicly because of the Swedish Secrecy Act. Data from the Total Population Register, Multi-Generation Register, National Patient Register, Cancer Register, Medical Birth Register, Longitudinal integrated database for health insurance and labour market studies were used for this study and made available by ethical approval. Researchers may apply for access through the Swedish Research Ethics Boards (http://www.etikprovningsmyndigheten.se) and from the primary data owners Statistics Sweden (http://www.scb.se) and the National Board of Health and Welfare (http://www.socialstyrelsen.se), in accordance with Swedish law.

## Abbreviations

CPRD: Clinical Research Practice Datalink
HES: Hospital Episodes Statistics
HR: Hazard Ratio
LISA: Longitudinal integrated database for health insurance and labour market studies
NPR: National Patient Register
