# Supplementary material for: Parental mental illness and the risk of offspring cancer in childhood: a pooled meta-analysis of English and Swedish national cohorts
Source: BMC Psychiatry. 2025 Oct 27;25:1022. doi: 10.1186/s12888-025-07520-w (PMC12557867; doi:10.1186/s12888-025-07520-w)
Supplement: Supplementary file 1 — Supplementary Material 1 [file 12888_2025_7520_MOESM1_ESM.docx]

Supplementary Files

**Parental mental illness and the risk of offspring cancer in childhood: a pooled meta-analysis of English and Swedish national cohorts**

Alicia Nevriana PhD^1^, Cemre Su Osam PhD^2^, Kyriaki Kosidou MD PhD^1,3^, Holly Hope PhD^2^, Darren M Ashcroft PhD^4,5^, Susanne Wicks PhD^1,3^, Christina Dalman MD PhD^1,3^, Kathryn M Abel PhD^2,6^, Matthias Pierce PhD^2^

Joint first author

Joint senior author

**Table S1** ICD codes used for identifying parental mental illness in the Swedish National Patient Register

**Table S2** ICD codes used for identifying childhood cancer diagnosis in England and Sweden

**Table S3** England (N=591,092) and Sweden (N=2,192,476) cohort demographics and health characteristics by parental mental illness exposure

**Table S4** Characteristics of children in Sweden separately by maternal (N=2,190,052) or paternal (N=2,160,740) mental illness

**Table S5** Distribution of children with maternal or paternal mental illness

**Supplementary Figure 1** Parental mental illness exposure ascertainment schematic

| **Table S1 ICD codes used for identifying parental mental illness in the Swedish National Patient Register** | | | | |
| --- | --- | --- | --- | --- |
| **Mental disorders diagnosis** | | **ICD-8** | **ICD-9** | **ICD-10** |
| Common mental illness | Depressive disorders, excluding those with psychotic symptoms | 300,40 | 300E; 311 | F32-34; F38-39; excluding F32.3 and F33.3 |
|  | Anxiety/stress-related disorders | 300; 305; 306,0; 306,9; 307;  excluding 300,40; 305,50 | 300A-D; 300F-H; 300W-X; 306; 307A; 308; 309 | F40-48 |
| Psychotic disorders | Non-affective psychotic disorders | 295; 297; 298;  excluding 295,70; 298,10 | 295; 297; 298; excluding 295H and 298B | F20-24; F28-29 |
|  | Affective psychotic disorders | 296; 295,70; 298,10 | 296; 295H; 298B | F25; F30-31; F32.3; F33.3 |
| Alcohol/substance use disorders | Alcohol/substance use disorders | 291; 294,30; 303; 304; excluding 303,00; 291,9 | 291; 292; 303; 304; excluding 291E; 292X | F10–16; F18-19; excluding 4^th^ digit .0 and .9 |
| Other | Eating disorders | 306,50; 305,50 | 307B; 307F | F50 |
|  | Personality disorders | 301 | 301 | F60-63; F68-69 |

| **Table S2 ICD codes used for identifying childhood cancer diagnosis in England and Sweden** | | |
| --- | --- | --- |
| **Cancer diagnosis** | **England (ICD-10)** | **Sweden (ICD-9)** |
| Lymphoid and haematopoietic | C81-C96 | 200-208 |
| Eye, brain, central nervous system | C69-C72 | 190-192 |
| Mesothelial and soft tissue | C45-C49 | 171 |
| Thyroid and other endocrine glands | C73-C75 | 193-194 |
| Bone and articular cartilage | C40-C41 | 170 |
| Others | C00-C26, C30-C39, C43-C44, C50-58, C60-C68, C76-C80, C97 | 140-165, 172-175, 179-189, 195-199 |

| **Table S3 England (N=591,092) and Sweden (N=2,192,476) cohort demographics and health characteristics by parental mental illness exposure** | | | | |
| --- | --- | --- | --- | --- |
| **Variables** | **England** | | **Sweden** | |
|  | **Children exposed to any maternal mental illness** | **Children unexposed to any maternal mental illness** | **Children exposed to any maternal or paternal mental illness** | **Children unexposed to any maternal or paternal mental illness** |
|  | **N=205,804** | **N=385,288** | **N=421,887** | **N=1,770,589** |
|  | n (%) | n (%) | n (%) | n (%) |
| **Region** | | | | |
| North East | 5,672 (2.8) | 6,979 (1.8) | - | - |
| North West | 34,629 (16.8) | 46,088 (12.0) | - | - |
| Yorkshire & The Humber | 7,403 (3.6) | 12,624 (3.3) | - | - |
| East Midlands | 7,206 (3.5) | 11,638 (3.0) | - | - |
| West Midlands | 24,080 (11.7) | 39,176 (10.2) | - | - |
| East of England | 23,540 (11.4) | 48,749 (12.7) | - | - |
| South West | 27,936 (13.6) | 43,730 (11.4) | - | - |
| South Central | 29,550 (14.4) | 50,981 (13.2) | - | - |
| London | 20,142 (9.8) | 75,957 (19.7) | - | - |
| South East Coast | 25,646 (12.5) | 49,366 (12.8) | - | - |
| **Antibiotic use during pregnancy** | | | | |
| Used | 74,641 (36.3) | 93,130 (24.2) | - | - |
| Not Used | 131,163 (63.7) | 292,158 (75.8) | - | - |
| **Maternal smoking^a^** | | | | |
| Never | 77,195 (37.5) | 201,257 (52.2) | 291,624 (72.0) | 1,372,415 (81.9) |
| Former | 23,732 (11.5) | 46,776 (12.1) | - | - |
| Current | 96,185 (46.7) | 90,891 (23.6) | 80,287 (19.8) | 167,444 (10.0) |
| Missing | 8,692 (4.2) | 46,364 (12.0) | 22,832 (5.8) | 83,953 (5.2) |
| **Parental history of cancer** | | | | |
| No | - | - | 407,978 (96.7) | 1,715,155 (96.9) |
| Yes | - | - | 13,909 (3.3) | 55,434 (3.1) |
| **Maternal comorbid depression or anxiety^b^** | | | | |
| No | - | - | 372,513 (88.3) | 1,736,689 (98.1) |
| Yes | - | - | 49,069 (11.6) | 31,781 (1.8) |
| Missing | - | - | 305 (0.1) | 2,119 (0.1) |
| **Maternal comorbid psychotic disorders^b^** | | | | |
| No | - | - | 414,528 (98.3) | 1,766,115 (99.8) |
| Yes | - | - | 7,054 (1.7) | 2,355 (0.1) |
| Missing | - | - | 305 (0.1) | 2,119 (0.1) |
| **Maternal comorbid other mental illness^b,c^** | | | | |
| No | - | - | 402,963 (95.5) | 1,757,544 (99.3) |
| Yes | - | - | 18,619 (4.4) | 10,926 (0.6) |
| Missing | - | - | 305 (0.1) | 2,119 (0.1) |
| **Paternal comorbid depression or anxiety^b^** | | | | |
| No | - | - | 390,776 (92.6) | 1,724,581 (97.4) |
| Yes | - | - | 26,795 (6.4) | 18,588 (1.1) |
| Missing | - | - | 4,316 (1.0) | 27,420 (1.6) |
| **Paternal comorbid psychotic disorders^b^** | | | | |
| No | - | - | 411,536 (97.6) | 1,740,830 (98.3) |
| Yes | - | - | 6,035 (1.4) | 2,339 (0.1) |
| Missing | - | - | 4,316 (1.0) | 27,420 (1.6) |
| **Paternal comorbid other mental illness^b,c^** | | | | |
| No | - | - | 398,836 (94.5) | 1,735,065 (98.0) |
| Yes | - | - | 18,735 (4.4) | 8,104 (0.5) |
| Missing | - | - | 4,316 (1.0) | 27,420 (1.6) |
| **Childhood cancer types** | | | | |
| Lymphoid and haematopoietic | 126 (40.1) | 254 (39.9) | 168 (35.1) | 1,906 (39.9) |
| Eye, brain, central nervous system | 70 (22.3) | 169 (26.5) | 129 (26.9) | 1,272 (26.7) |
| Mesothelial and soft tissue | 9 (2.9) | 40 (6.3) | 29 (6.1) | 382 (8.0) |
| Thyroid and other endocrine glands | 14 (4.5) | 34 (5.3) | 36 (7.5) | 245 (5.1) |
| Bone and articular cartilage | 20 (6.4) | 22 (3.5) | 30 (6.3) | 155 (3.3) |
| Others | 75 (23.9) | 118 (18.5) | 87 (18.2) | 812 (17.0) |
| ^a^England: Any time during child’s life (from the beginning of pregnancy till child’s end of follow up). Sweden: At first antenatal visit, for children born 1991-2010. | | | | |
| ^b^Measured at any time point up until childbirth | | | | |
| ^c^Including alcohol/substance use disorders, eating disorders, personality disorders | | | | |

| **Table S4 Characteristics of children in Sweden separately by maternal (N=2,190,052) or paternal (N=2,160,740) mental illness** | | | | |
| --- | --- | --- | --- | --- |
| **Variables** | **Children exposed to any maternal mental illness** | **Children unexposed to any maternal mental illness** | **Children exposed to any paternal mental illness** | **Children unexposed to any paternal mental illness** |
|  | **N=276,120** | **N=1,913,932** | **N=194,378** | **N=1,966,362** |
|  | **n (%)** | **n (%)** | **n (%)** | **n (%)** |
| **Follow up time (year)** | | | | |
| Mean (SD) | 13.7 (4.4) | 13.1 (5.0) | 13.8 (4.4) | 13.2 (4.9) |
| **Child sex** | | | | |
| Female | 133,083 (48.2) | 931,640 (48.7) | 94,597 (48.7) | 955,840 (48.6) |
| Male | 143,037 (51.8) | 982,292 (51.3) | 99,781 (51.3) | 1,010,522 (51.4) |
| **Birth year** | | | | |
| 1991-1995 | 61,952 (22.4) | 515,805 (27.0) | 49,063 (25.2) | 523,658 (26.6) |
| 1996-2001 | 78,778 (28.5) | 467,900 (24.5) | 56,668 (29.2) | 485,061 (24.7) |
| 2002-2007 | 84,498 (30.6) | 530,964 (27.7) | 56,727 (29.2) | 551,629 (28.1) |
| 2008-2013 | 50,892 (18.4) | 399,263 (20.9) | 31,920 (16.4) | 406,014 (20.7) |
| **Parental country of birth** | | | | |
| All known parents born in Sweden | 198,424 (71.9) | 1,437,563 (75.1) | 134,123 (69.0) | 1,483,487 (75.4) |
| Some parents born outside Sweden | 77,695 (28.1) | 476,335 (24.9) | 60,255 (31.0) | 482,851 (24.6) |
| Missing | 1 (0.0) | 34 (0.0) | 0 (0.0) | 24 (0.0) |
| **Maternal age at delivery (year**)**^a^** | | | | |
| <20 | 8,299 (3.0) | 21,361 (1.1) | 6,275 (3.2) | 22,176 (1.1) |
| 20-29 | 135,422 (49.0) | 849,466 (44.4) | 96,534 (49.7) | 874,956 (44.5) |
| 30-39 | 122,134 (44.2) | 974,493 (50.9) | 84,104 (43.3) | 999,135 (50.8) |
| >40 | 10,265 (3.7) | 68,612 (3.6) | 7,416 (3.8) | 69,564 (3.5) |
| **Paternal age at delivery (year)^b^** | | | | |
| <20 | 2,403 (0.9) | 5,502 (0.3) | 2,025 (1.0) | 5,899 (0.3) |
| 20-29 | 95,159 (34.5) | 542,966 (28.4) | 65,256 (33.6) | 572,830 (29.1) |
| 30-39 | 137,398 (49.8) | 1,086,200 (56.8) | 97,395 (50.1) | 1,125,707 (57.3) |
| >40 | 37,411 (13.6) | 254,470 (13.3) | 29,702 (15.3) | 261,926 (13.3) |
| **Parental education** | | | | |
| Compulsory | 30,562 (11.1) | 104,300 (5.5) | 25,399 (13.1) | 103,556 (5.3) |
| Secondary | 141,964 (51.4) | 851,194 (44.5) | 102,619 (52.8) | 879,546 (44.7) |
| University | 97,727 (35.4) | 921,880 (48.2) | 62,249 (32.0) | 949,759 (48.3) |
| Missing | 5,867 (2.1) | 36,558 (1.9) | 4,111 (2.1) | 33,501 (1.7) |
| **Household disposable income in quintiles** | | | | |
| Q1 (most deprived) | 53,890 (19.5) | 282,731 (14.8) | 43,111 (22.2) | 278,604 (14.2) |
| Q2 | 80,644 (29.2) | 504,635 (26.4) | 56,390 (29.0) | 523,632 (26.6) |
| Q3 | 64,869 (23.5) | 466,228 (24.4) | 45,208 (23.3) | 483,244 (24.6) |
| Q4 | 42,233 (15.3) | 365,455 (19.1) | 27,764 (14.3) | 378,154 (19.2) |
| Q5 (least deprived) | 31,382 (11.4) | 276,815 (14.5) | 19,838 (10.2) | 286,387 (14.6) |
| Missing | 3,102 (1.1) | 18,068 (0.9) | 2,067 (1.1) | 16,341 (0.8) |
| **Maternal smoking at first antenatal visit^e^** | | | | |
| No | 187,579 (70.9) | 1,476,448 (81.4) | 132,612 (70.7) | 1,519,040 (81.3) |
| Yes | 54,829 (20.7) | 192,902 (10.6) | 39,707 (21.2) | 204,765 (11.0) |
| Missing | 15,260 (5.9) | 91,523 (5.2) | 10,409 (5.7) | 94,907 (5.2) |
| **Parental history of cancer** | | | | |
| No | 266,888 (96.7) | 1,853,830 (96.9) | 188,053 (96.8) | 1,903,991 (96.8) |
| Yes | 9,232 (3.3) | 60,102 (3.1) | 6,325 (3.3) | 62,371 (3.2) |
| **Maternal comorbid depression or anxiety^c^** | | | | |
| No | 231,274 (83.8) | 1,877,928 (98.1) | 180,388 (92.8) | 1,899,269 (96.6) |
| Yes | 44,846 (16.2) | 36,004 (1.9) | 13,685 (7.0) | 64,974 (3.3) |
| Missing | 0 (0.0) | 0 (0.0) | 305 (0.2) | 2,119 (0.1) |
| **Maternal comorbid psychotic disorders^c^** | | | | |
| No | 269,408 (97.6) | 1,911,235 (99.9) | 192,251 (98.9) | 1,956,966 (99.5) |
| Yes | 6,712 (2.4) | 2,697 (0.1) | 1,822 (0.9) | 7,277 (0.4) |
| Missing | 0 (0.0) | 0 (0.0) | 305 (0.2) | 2,119 (0.1) |
| **Maternal comorbid other mental illness^c,d^** | | | | |
| No | 259,285 (93.9) | 1,901,222 (99.3) | 187,206 (96.3) | 1,942,518 (98.8) |
| Yes | 16,835 (6.1) | 12,710 (0.7) | 6,867 (3.5) | 21,725 (1.1) |
| Missing | 0 (0.0) | 0 (0.0) | 305 (0.2) | 2,119 (0.1) |
| **Paternal comorbid depression or anxiety^c^** | | | | |
| No | 261,277 (94.6) | 1,851,754 (96.8) | 171,297 (88.1) | 1,944,060 (98.9) |
| Yes | 10,527 (3.8) | 34,758 (1.8) | 23,081 (11.9) | 22,302 (1.1) |
| Missing | 4,316 (1.6) | 27,420 (1.4) | 0 (0.0) | 0 (0.0) |
| **Paternal comorbid psychotic disorders^c^** | | | | |
| No | 269,863 (97.7) | 1,880,091 (98.2) | 188,756 (97.1) | 1,963,610 (99.9) |
| Yes | 1,941 (0.7) | 6,421 (0.3) | 5,622 (2.9) | 2,752 (0.1) |
| Missing | 4,316 (1.6) | 27,420 (1.4) | 0 (0.0) | 0 (0.0) |
| **Paternal comorbid other mental illness^c,d^** | | | | |
| No | 263,526 (95.4) | 1,867,984 (97.6) | 177,791 (91.5) | 1,956,110 (99.5) |
| Yes | 8,278 (3.0) | 18,528 (1.0) | 16,587 (8.5) | 10,252 (0.5) |
| Missing | 4,316 (1.6) | 27,420 (1.4) | 0 (0.0) | 0 (0.0) |
| **Childhood cancer types** | | | | |
| Lymphoid and haematopoietic | 112 (35.1) | 1,958 (39.8) | 67 (33.0) | 1,986 (39.7) |
| Eye, brain, central nervous system | 81 (25.4) | 1,320 (26.8) | 60 (29.6) | 1,329 (26.6) |
| Mesothelial and soft tissue | 23 (7.2) | 388 (7.9) | 8 (3.9) | 399 (8.0) |
| Thyroid and other endocrine glands | 24 (7.5) | 255 (5.2) | 18 (8.9) | 261 (5.2) |
| Bone and articular cartilage | 22 (6.9) | 163 (3.3) | 12 (5.9) | 172 (3.4) |
| Others | 57 (17.9) | 842 (17.1) | 38 (18.7) | 851 (17.0) |
| ^a^Including children with known mothers | | | | |
| ^b^Including children with known fathers | | | | |
| ^c^Measured at any time point up until childbirth | | | | |
| ^d^Including alcohol/substance use disorders, eating disorders, personality disorders | | | | |
| ^e^Only among children born 1991-2010 | | | | |

| **Table S5 Distribution of children with maternal or paternal mental illness^a^** | | | |
| --- | --- | --- | --- |
| **Type of mental illness^b^** | **England** | **Sweden** | |
|  | **Maternal** | **Maternal** | **Paternal** |
|  | **N=591,092** | **N=2,190,052** | **N=2,160,740** |
|  | **n (%)** | **n (%)** | **n (%)** |
| Any mental illness | 205,804 (34.8) | 276,120 (12.6) | 194,378 (9.0) |
| Depression or anxiety | 202,948 (34.3) | 258,085 (11.8) | 158,564 (7.3) |
| Psychotic disorders | 4,095 (0.7) | 34,950 (1.6) | 24,749 (1.2) |
| Alcohol/substance use disorders | 6,854 (1.2) | 27,912 (1.3) | 59,483 (2.8) |
| Other mental illness | 6,105 (1.0) | 32,032 (1.5) | 16,764 (0.8) |
| ^a^Measured from 1 year before birth until the end of follow up | | | |
| ^b^Categories are not mutually exclusive | | | |


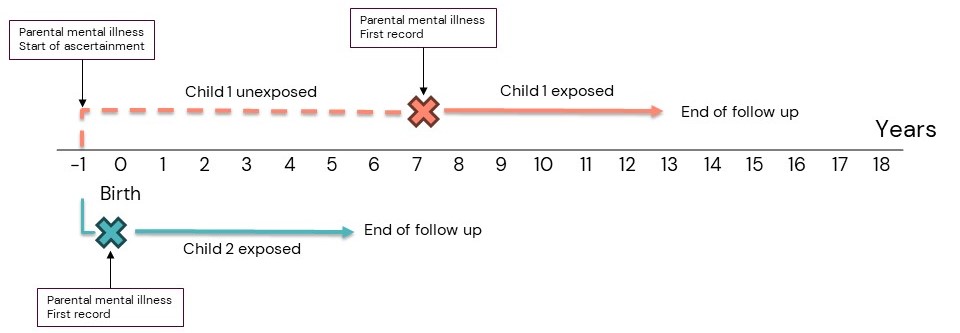


Supplementary Figure 1 Parental mental illness exposure ascertainment schematic
